# The Enigma of Bioactivity and Toxicity of Botanical Oils for Skin Care

**DOI:** 10.3389/fphar.2020.00785

**Published:** 2020-05-29

**Authors:** Erin M. Moore, Charles Wagner, Slavko Komarnytsky

**Affiliations:** ^1^Plants for Human Health Institute, North Carolina State University, Kannapolis, NC, United States; ^2^Department of Food, Bioprocessing, and Nutrition Sciences, North Carolina State University, Raleigh, NC, United States; ^3^Department of Biology, Catawba College, Salisbury, NC, United States; ^4^Department of Plant and Microbial Biology, North Carolina State University, Raleigh, NC, United States

**Keywords:** natural oils, skin barrier function, skin aging, wound healing, atopic dermatitis, for skin care

## Abstract

Botanical oils have a long history of traditional use and are routinely applied to skin care. The focus of this review is to contrast the functionality of skin oils *versus* the differential biological and toxicological effects of major plant oils, and to correlate them to their compositional changes. In total, over 70 vegetable oils were clustered according to their lipid composition to promote awareness of health practitioners and botanical product manufacturers for the safety and efficacy of oil-based interventions based on their fatty acid profiles. Since multiple skin disorders result in depletion or disturbance of skin lipids, a tailored mixture of multiple botanical oils to simultaneously maintain natural skin-barrier function, promote repair and regeneration of wounded tissues, and achieve corrective modulation of immune disorders may be required. As bioactive constituents of botanical oils enter the human body by oral or topical application and often accumulate in measurable blood concentrations, there is also a critical need for monitoring their hazardous effects to reduce the possible over-added toxicity and promote maximal normal tissue sparing. The review also provides a useful tool to improve efficacy and functionality of fatty acid profiles in cosmetic applications.

## Introduction

Botanical oils are lipids or fats derived from one or more plant parts and can be broadly classified into fixed (vegetable) and essential (volatile) oils. Four agricultural crops (oil palm, soybeans, rapeseed, and sunflower) serve as major sources of fixed oil for nutritional applications (Dyer et al., 2008). These oils are a combination of saturated (no double bonds), monounsaturated (one double bond), and polyunsaturated (two or more double bonds) fatty acids of varying carbon chain length, attached to a glycerol molecule. Natural mono-(MUFA) and poly-(PUFA) unsaturated oils contain double bonds in the less thermodynamically stable *cis* configuration that is prone to oxidative deterioration. As such, unsaturated oils can be industrially processed to remove (saturate) double bonds by partial hydrogenation. This process, however, introduces *trans* configuration into the fatty acids that has detrimental health effects (Ascherio, 2006). The degree of fatty acid saturation defines the fluidity, molecular packing, lipoxidative damage, and integrity of cell membranes (Pamplona, 2008).

As complex bioactive constituents in botanical oils coevolved to mediate plant-animal interactions, they likely contain functional, biologically relevant chemical spaces and pharmacophores that were selected to interact with animal and human cell targets (Sharifi-Rad et al., 2017). Oils are widely used to prevent or ameliorate human diseases, especially in the form of topical applications to promote skin health, heal injuries and burns, decrease scarring, improve cosmetic outcomes, reduce social stigmatizing, and promote wellbeing (Vaughn et al., 2018). Plant oils can rapidly penetrate through the lipid structures of the skin and interact with the cell membrane proteins to induce their conformational modifications (Herman and Herman, 2015). Their unique physiochemical properties are utilized for natural enhancement of skin penetration during transdermal drug delivery (Edris, 2007).

This review therefore summarizes recent evidence on utility of botanical oils for topical skin care, including maintenance of natural skin-barrier function, repair and regeneration of wounded tissues, and modulation of immune skin disorders. As bioactive constituents of oils achieve measurable blood concentrations following the oral absorption, skin penetration, or inhalation routes, there is a need for monitoring hazardous effects of these bioactive and their metabolites to reduce the possible over-added toxicity and promote maximal normal tissue sparing.

## Structure and Function of the Skin

The skin functions to maintain temperature and hydration, while protecting the body from environmental injuries and microbial infections. Damaged skin allows entry of chemical irritants, microorganisms, and allergens that promote and amplify skin inflammatory and immune responses (Chen and DiPietro, 2017).

Skin consists of two major structural layers, the epidermis with embedded sebaceous glands, hair follicles, and sweat glands (epithelium) and the dermis (a mixture of loose and dense connective tissues). While the epidermis consists predominantly of continuously replenished and shed, terminally differentiated keratinocytes, the dermis is populated by a variety of cell types including fibroblasts, immune cells, and white adipocytes surrounded by the fibrillar collagen. The basement membrane comprised of type IV collagen and laminin separates these two layers and serves as an anchor for dermal papillae and the smooth muscles that control hair follicles (Watt and Fujiwara, 2011). The epithelial layer is also colonized by neural crest-derived melanocytes responsible for melanin production (skin color and UV protection) and dendritic immune antigen-presenting cells (monocyte-derived Langerhans and thymus-derived T cells) that respond to injury or infection with the production of proinflammatory cytokines, thus maintaining the innate immune response of the skin (Ohteki and Koyasu, 2001).

The upper epidermis (*stratum corneum*) also contains keratohyalin protein granules and lamellar lipid bodies that prevent water loss. The major proteins of *stratum corneum* are type I (acidic) and type II (basic) keratins, filaggrin (proteolytically cleaved to release the amino acids as a moisturizing factor), loricrin and involucrin (cross-linking factors), and small proline rich proteins SPRs (Wickett and Visscher, 2006). Main components of lamellar lipid bodies are ceramides (sphingolipids linked to long-chain fatty acids, 50%), cholesterol (25%), and free fatty acids (cleaved from keratinocyte membrane phospholipids, 15%) that maintain the acidic skin surface pH of 4.0–5.5 and the diversity of skin microbiome (Elias and Choi, 2005). Cosmetic-grade glycerin (a natural component of triglyceride lipids) and petroleum-distilled mineral oil (Baby Oil as branded by Johnson & Johnson) or petrolatum (Vaseline as branded by Unilever) are effective skin-conditioning agents that increase hydration and improve elasticity of the epidermis (Rawlings and Lombard, 2012).

The dermis contains stromal cells, with fibroblasts making up the major cell type. Structural cells of the peripheral nervous, immune, vascular, and lymph systems either reside or temporarily migrate through the skin (Rognoni and Watt, 2018). Upper papillary (proliferative) and lower reticular (secretory) dermal layers are separated by the vascular plexus. Fibroblast residing in each layer are epigenetically modified to either proliferate or secrete extracellular matrix (ECM) (Collins et al., 2011). Type I, III, and V collagens are the most abundant fibrillary ECM proteins. Additional fibril-associated collagens connect collagen I and III fibrils with decorin and perlecan proteoglycans (Reed and Iozzo, 2002). The elastic fiber network and glycosaminoglycans such as hyaluronic acid allow for functional interaction and capture of water to generate osmotic pressure responsible for skin turgor (Juhlin, 1997). As the dermis is critical in maintenance of healthy and repair of wounded skin by means of functional and nutritional support of the epidermis (Waller and Maibach, 2006), it often serves as a primary target for therapeutic and cosmetic interventions targeting collagen and elastin production, or cellular responses within the dermal tissue (Badenhorst et al., 2014).

### Lipids of Healthy Skin

Skin surface lipids derived from the epidermis and sebaceous glands are found in decreasing order on scalp > face > back > chest > abdomen > arms > legs > palms and soles. The latter do not contain sebaceous glands but receive small amounts of carryover lipids from other areas of the body (Downing and Strauss, 1974). Human sebaceous glands are a unique source of wax esters and squalene (**Table 1**). The composition of human skin lipids also differs from that of other mammals by higher content of triacylglycerols and free fatty acids (Cheng and Russell, 2004). Fatty acids naturally present in human *stratum corneum* are mostly saturated docosanoic acid 22:0, lignoceric acid 24:0, and hexacosanoic acid 26:0 that are often branched, methylated, and/or hydroxylated, although smaller quantities of oleic acid 18:1(n-9) and linoleic acid 18:2(n-6) have been also reported (Vicanová et al., 1997; Bouwstra and Honeywell-Nguyen, 2002). The perceived oiliness of the skin, however, does not depend on total surface lipids nor the proportion of free fatty acids, but rather correlates with the larger ratios of unsaturated fatty acids and wax esters in the sebum.

**Table 1:** Lipid class composition of various skin sites (% total lipid).

Lipid class^1^	Human skin surface					Other mammals	
	Sebum	Scalp	Face	Forehead	Back	Mouse	Sheep
***Basic analysis***							
Squalene	12	12–14	12	12	12–16	–	–
Wax esters	26	21–23	23	26	22–23	5	10
Fatty acids (total)	58	56–65	65	62	61–66	6	–
							
***Extended analysis***							
Squalene	12	12–13	12	12	11–16	–	–
Wax esters	26	20–22	23	25	22	5	10
Triglycerides (bound)	–	29–32	35	43	43–46	6	–
Fatty acids (free)	–	29–33	27	16	16	–	–
Sterol esters	3	3	3	2	3	10	46
Sterols	2	2	1	1	1-2	13	12
Diesters	–	–	–	–	–	65	21
Hydrocarbons	–	1	1	–	1-2	–	–

^1^Adapted with modifications from (Downing and Strauss, 1974).

As the most abundant saturated fatty acid in human sebum, palmitic acid 16:0 is metabolized by both FADS2 and SCD desaturases to sapienic acid 16:1(n-10) and sebaleic acid 18:2(n-10). Desaturation of stearic acid 18:0 by the SCD enzyme also results in accumulation of oleic acid 18:1(n-9) in human sebum (Park et al., 2016). Small amounts of two essential fatty acids, linoleic acid 18:2(n-6), and α-linolenic acid 18:3(n-3), as well as a conditionally essential arachidonic acid 20:4(n-6) that becomes essential if a deficiency in linoleic acid develops, are also found in human sebum (**Table 2**).

**Table 2:** Fatty acid composition from various body sites (%).

Fatty acid	Scalp^1^	Sole^2^		Forearm^3^			Erythrocyte^4^	Plasma^5^
	Surface	Live	SC	N	PU	PI	Membrane	Lipids (μmol/L)
Lauric	0.1–1.9	*t*–0.1	0.2	nr	nr	nr	–	–
Myristic	4–8	1.4–2.5	1.1–1.9	1.1	1.5	0.8	–	16.2–325.7
Palmitic	18–29	40.6–48.6	24.6–25.1	14	13.9	12	23–26	285.4–4064.5
Palmitoleic	–	–	–	2.3	2.6	3.9	–	27.7–555.9
Sapienic		–	–				nr	–
Stearic	2–5	33.9–34.8	18.6–19.3	11.1	10.9	10.1	14–21	110.2–1013.7
Oleic	11–19	86.2–86.0	83.6–80.0	15.1	13.7	16.8	14–19	178.7–3210.5
Linoleic	1–2	96.7	96.1–96.7	21.5	21	15.7	11–12	279.7–4970.5
α-Linolenic	–	–	–	nr	nr	nr	0.1–0.3	–
Arachidic	0.2–1.8	1.0–1.2	3.9–4.8	1.6	1.7	1.8	–	*t*–29.8
Mead	–	–	–	1.5	1.4	1.4	1–2	–
Arachidonic	–	–	–	6.2	6.5	5.0	14–16	42.7–882.8
EPA	–	–	–	nr	nr	nr	1	4.4–215.4
Behenic	0.1–1.2	1.2–2.9	7.5–7.8	2.7	2.9	1.4	–	*t*–39.0
DPA	–	–	–	nr	nr	nr	2–3	*t*–88.5
DHA	–	–	–	nr	nr	nr	4–7	7.2–237.5
Lignoceric	0.3–1.2	1.0–3.7		10	10.5	4.2	–	*t–*15.7

Adapted with modifications from:

^1^Scalp skin surface (Koch et al., 1982);

^2^Sole skin epidermis (Ansari et al., 1970);

^3^Normal (N), uninvolved (PU), and involved (PI) psoriatic forearm skin (Chapkin et al., 1986);

^4^Erythrocyte membrane (Akinyemi et al., 2017);

^5^Plasma lipids (Abdelmagid et al., 2015).

The extracutaneous traffic of lipids into the epidermis plays a significant role in permeability barrier formation. Dietary fatty acids (Reynolds et al., 1978), sterols (Bhattacharyya et al., 1983), and glucosylceramides (Tsuji et al., 2006) traffic through the extracutaneous tissues to contribute to the epidermal lipid pool. Antimicrobial lauric acid 12:0 and sapienic acid 16:1(n-10), antifungal caprylic acid 8:0 and capric acid 10:0, and antioxidant vitamin E are delivered to the skin surface to naturally reduce oxidative damage and provide basic antimicrobial defenses (Fischer et al., 2014). The epidermis also lacks d6 and d5 desaturase activity and imports arachidonic acid 20:4(n-6) from the extraepidermal sites (Chapkin and Ziboh, 1984). Finally, essential fatty acids critical for the efficient structure and function of the skin must be also delivered from the diet and incorporated into ceramides (Kendall et al., 2017) (**Table 3**). Deficiency of essential fatty acids leads to scaliness of the skin and an increased water consumption, mainly due to disruption of the water permeability barrier and an increase in trans-epidermal water loss (Basnayake and Sinclair, 1956). Linoleic acid 18:2(n-6) is also selectively targeted for β-oxidation by the sebaceous cells as a unique energy source for their function (Pappas et al., 2002), while application of nicotinamide (Tanno et al., 2000) and L-lactic acid (Rawlings et al., 1996) produce similar effects.

**Table 3:** Compositions of skin bound fatty acids by various lipid class (%).

Fatty acid^1^	Lipid number	Free	Ester-linked		Long-chain bases		Amide-linked		Cholesteryl esters
			Cer 1	Cer 6I	Cer 2	Cer 3	Cer 2	Cer 6I	
Myristic	14:0	0.8	2	–	–	–	0.2	–	–
Myristoleic	14:1	–	2.3	–	–	–	–	–	–
Palmitic	16:0	7.4	18.0	30.2	0.6	3.1	3.6	1.7	9.9
Palmitoleic	16:1(n-7)	0.7	4.8	–	0.7	–	–	–	3.0
Margaric	17:0	0.8	1.2	–	2.1	8.0	0.4	1.4	1.1
Stearic	18:0	9.1	9.1	4.8	11.7	12.8	4.4	9.9	4.6
Oleic	18:1(n-9)	5.7	11.6	–	35.6	–	–	–	68.2
Linoleic	18:2(n-6)	1.4	20.7	–	–	–	–	–	–
Nonadecylic	19:0	1.1	0.1	–	1.6	16.0	1.0	1.7	–
Arachidic	20:0	5.9	3.5	3.6	6.5	14.4	3.8	1.6	6.6
Heneicosylic	21:0	1.9	0.2	3.1	20.7	10.1	1.2	1.4	1.1
Behenic	22:0	15.3	4.8	3.3	1.5	21.4	8.7	1.7	1.3
Tricosylic	23:0	6.2	–	–	–	2.1	5.7	1.3	1.0
Lignoceric	24:0	26.9	8.4	20.2	10.5	5.1	30.4	27.9	1.4
Pentacosylic	25:0	5	1.8	6.3	–	2.1	7.8	11.3	1.1
Cerotic	26:0	8.5	4.1	18.5	–	4.9	18.3	34.8	1.1

^1^Adapted with modifications from (Wertz et al., 1987).

### Lipids in Skin Diseases and Wound Healing

External damage by physical (mechanical injury, UV-irradiation, heat, excessive moisture, pressure, or friction), chemical (solvents, irritants, or allergens) or microbial assaults (bacteria, fungi, or viruses) results in injuries in the form of wounds, burns, calluses, or scars. Dry, cracked, or fissured skin is often presented with major changes in its lipid profile, resulting in excessive water loss and direct exposure to allergens and microbes that further irritate and inflame skin.

Injured skin heals in four overlapping stages that include hemostasis (blood clot formation), inflammation (infiltration of immune cells), proliferation (angiogenesis, granulation, epithelialization, and ECM remodeling by proliferating and migrating fibroblasts and keratinocytes), and maturation (wound contraction and resolution of inflammation) of skin layers. When these processes are disturbed due to an underlying genetic or clinical disorder, the healing pathology results in an ulcerative chronic wound, a hypertrophic scar, or a keloid (Eming et al., 2014). Proinflammatory cytokines and lipid mediators synthesized and released by neutrophils at the site of the wound must be tightly regulated and resolved, otherwise leading to persistent inflammation and uncontrolled proliferation and collagen secretion by skin cells. This pathology directly interferes with contraction of the wound that comprises of proliferation and migration of keratinocytes into the wounded area, and differentiation of fibroblasts into myofibroblasts (Leoni et al., 2015). While some organisms are fully capable of repairing and regenerating injured tissues, in humans this process occurs only in fetal skin and is partially preserved in the gut epithelium and hematopoietic system (Lorenz et al., 1992).

Direct supplementation or replacement of skin lipids can be explored in the prevention or treatment of skin pathologies. Lipid-based barrier repair creams like EpiCeram by Promius Pharma (ceramides, cholesterol, and free fatty acids, 3:1:1), Lipobase by Astellas Pharma (sorbitan oleate, carnauba wax, ceramide 3, oleic acid, palmitic acid, and cholesterol), CeraVe by L’Oreal (ceramides 6II, 3, 1, phytosphingo-sine, hyaluronic acid), Triceram by Dentaurum (lanolin, ceramides, soybean sterol, linoleic acid, hyaluronic acid), Atopiclair by Alliance Pharma (glycyrrhetinic acid 2%, hyaluronic acid, grapevine extract, telmestine, shea butter), and MimyX by Stiefel Laboratories (a cannabinoid agonist N-palmitoylethanolamine, olive oil, palm glycerides, vegetable oil, squalane) have been cleared for marketing by the FDA as 510(k) medical devices with no defined active ingredients. The synthetic sebum mixture consisting of 45% triglycerides, 25% wax monoesters (jojoba oil), 17% fatty acids, and 12% squalene has been also proposed (Wertz, 2009). Additional mixtures of unsaturated fatty acids that modulate skin proliferative and immune responses may also promote wound closure by direct effects on skin inflammation and permeability (Cardoso et al., 2004).

The majority of skin diseases also present with variation or depletion of the major skin lipids as reported for atopic dermatitis (reduced ceramides and C20–26 fatty acids), psoriasis (reduced ceramides), type 2 Gaucher disease (increased glucosylceramides), acne vulgaris (reduced sphingolipids), atopic eczema (increased short-chain ceramides), and aged dry skin (reduced ceramides) (Sahle et al., 2015). Pathological microbial infection of the skin also alters skin lipid profiles as shown for *Propionibacterium* infections observed in acne (Saint-Leger et al., 1986) and *Pityrosporum* infections associated with seborrheic dermatitis (Bergbrant et al., 1991). Stress and other physiological factors often exacerbate skin conditions and healing processes by changes in neurohormone and steroid hormone levels that directly affect blood flow, metabolic and immune status of the skin, and function of hair follicles (Hunter et al., 2015).

## Approach

PubMed, Scopus, Google Scholar, ScienceDirect, JSTOR, and National Center for Biotechnology Information (NCBI) were used to find and review relevant research articles. The plantlist.org was used for aligning Scientific Binomials. All major botanical fixed oils were clustered based on their fatty acid profiles and sources. The individual fatty acids were discussed according to their skin care potential, with a specific distinction toward their applicability to different and often opposing biological outcomes. All presented profiles are averages described in multiple publications, as cultivar, geographical, and ecological factors show considerable variation.

## Botanical Oils for Topical Skin Care

Inexpensive and readily available botanical oils are routinely used for topical skin applications. They may enhance skin function by forming a physical barrier, supplying fatty to different skin layers, activating peroxisome proliferator-activated receptor-α (PPAR-α) signaling, or decreasing cutaneous inflammation (De Luca and Valacchi, 2010). Chemical diversity found in botanical oils leads to a variety of pharmacological activities and modes of action depending on quantities and proportions of individual chemical constituents in these complex mixtures. In general, it appears high linoleic acid 18:2(n-6) containing botanical oils (i.e., sunflower) are more beneficial to skin health (Hanley et al., 1998) when compared to the high oleic acid 18:1(n-9) counterparts (Jiang et al., 2000). Therefore, different ratios of individual fatty acids present in botanical oils often result in opposite, either beneficial or detrimental, effects on epidermal barrier function and comedogenicity and merit detailed inquiry (Darmstadt et al., 2002).

### Saturated Fatty Acids

Fixed oils of botanical origin (vegetable oils) are complex mixtures of triacylglycerols (fatty acid esters of glycerol) with some minor components such as tocopherols, phytosterols, and polyphenols that are either cold-pressed (virgin oils), solvent extracted from oil-containing plant parts, or mechanically separated from the aqueous phase after crushing. Dietary vegetable oils are also for the most part refined to produce a bland, stable oil for consumption. Saturated fatty acids present in fixed oils do not contain double bonds and are found mostly as white solids under normal conditions.

#### Caprylic Acid 8:0 and Capric Acid 10:0

These saturated fatty acids are found in high quantities in goat milk, but also present as minor constituents of coconut oil and palm kernel oil. Large quantities of these fatty acids are only found in seed oils of several species of the genus *Cuphea*, while capric acid also dominates the fatty acid profile of elm *Ulmus americana* L. seed oil. Known for their antimicrobial properties (Valipe et al., 2011), both molecules play predominantly formulation roles in cosmetic applications by decreasing the melting point, lowering viscosity, providing efficient solvent, oxidative resistance, emollient, and conditioning properties to skin products (Chaudhuri and Bojanowski, 2017).

#### Lauric Acid 12:0 and Myristic Acid 14:0

Lauric acid 12:0, as a component of triglycerides, is a major fatty acid present in coconut, palm, laurel, babassu, murumuru, and ucuhuba oil and butter. Lauric acid 12:0 reacted with sodium hydroxide produces laurate salts that form the basis for soap production. Among these plant oils, laurel fruit oil is unique due to its low saturation ratio (42–45%) compared to the other oils in this group (80–90%). Additionally, nutmeg, and ucuhuba butters have a unique saturated fatty acid profile that is dominated by myristic acid 14:0 (**Table 4**). Both lauric acid 12:0 and myristic acid 14:0 showed a moderate degree of bacteriostatic properties, with the former one being also bactericidal at the MBC range of 7–375 μg/ml (Fischer et al., 2012). Lauric acid 12:0 is also reaching the skin surface naturally as a part of the outward sebum flow that is dominated by the palmitoleic acid 16:1(n-7) isoforms (Weitkamp et al., 1947), and exerts moderate inhibitory effects on the growth of skin bacteria associated with inflammatory acne at the minimal inhibitory concentration (MIC) range of 1–4 μg/ml (Nakatsuji et al., 2009). Among these plant oils, only coconut (Strunk et al., 2018) and palm kernel (Chiabi et al., 2011) oils have been studied clinically for skin care, especially that of neonates. Even though these oils were noted as good emollients that prevent transdermal water loss and increase skin moisture, controversy subsists about other beneficial effects associated with their use. As topically applied botanical oils penetrate largely only into the upper layers of the epidermis (Patzelt et al., 2012), their application occludes the skin surface and leads to break outs on most skin types, other than very dry skin, due to their high comedogenic properties.

**Table 4:** Botanical fixed oils clustered according to their fatty acid composition and saturation SFA:MUFA:PUFA ratios.

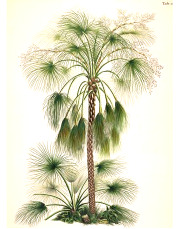	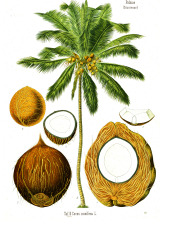	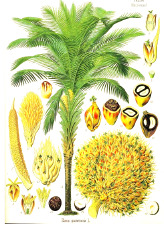	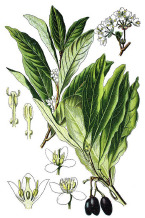	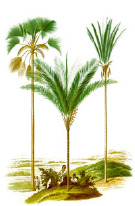	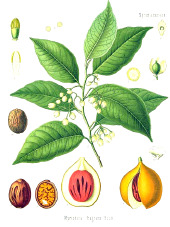	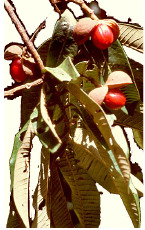	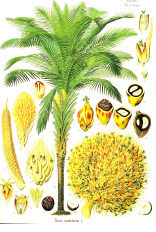	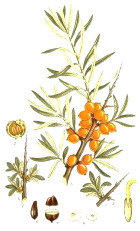	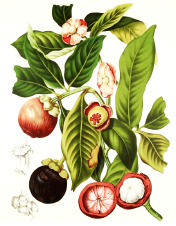
Murumuru seed	Coconut kernel	Palm kernel	Laurel fruit	Babassu seed	Nutmeg nut	Ucuhuba seed	Palm pulp	Buckthorn fruit	Kokum seed
*Astrocaryum murumuru* Mart.	*Cocos nucifera* L.	*Elaeis guineensis* Jacq.	*Laurus nobilis* L.	*Attalea speciosa* Mart.	*Myristica fragrans* Houtt.	*Virola surinamensis* (Rol. ex Rottb.) Warb.	*Elaeis guineensis* Jacq.	*Hippophae rhamnoides* L.	*Garcinia indica* (Thouars) Choisy
90:7:3	93:6:2	82:16:3	48:37:15	80:17:3	88:8:2	93:4:1	50:39:11	28:47:25	61:38:1
Lauric	Lauric	Lauric	Lauric	Lauric	Myristic	Myristic	Palmitic	Palmitic	Stearic
49%	48%	46%	43%	34%	79%	71%	44%	27%	59%
Myristic	Myristic	Myristic	Oleic	Myristic	Oleic	Lauric	Oleic	Palmitoleic	Oleic
30%	19%	18%	37%	19%	7%	16%	39%	25%	38%
Palmitic	Palmitic	Oleic	Linoleic	Oleic	Palmitic	Palmitic	Linoleic	Linoleic	Palmitic
7%	9%	16%	15%	17%	6%	4%	10%	16%	2%
Oleic	Caprylic	Palmitic	Palmitic	Palmitic	Lauric	Oleic	Stearic	Oleic	Linoleic
7%	8%	8%	5%	11%	2%	4%	4%	15%	1%
Linoleic	Capric	Capric	Myristic	Capric	Linoleic	Linoleic	Myristic	Linolenic	
3%	7%	4%	0.1%	6%	1%	1%	1%	9%	
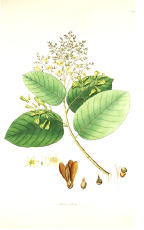	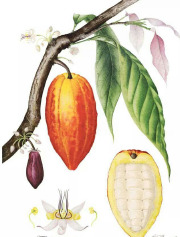	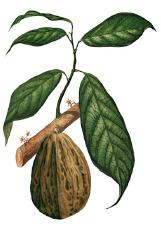	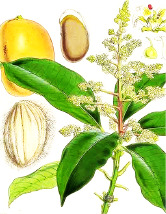	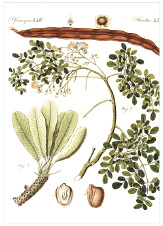	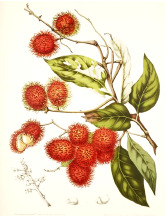	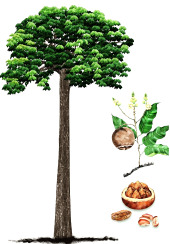	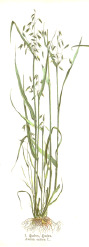	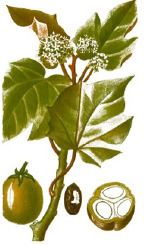	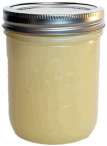
Sal fruit	Cocoa bean	Cupuacu bean	Mango seed	Shea nut	Rambutan seed	Brazil nut	Oat seed	Jatropha seed	Tallow fat
*Shorea robusta* C.F.Gaertn.	*Theobroma cacao* L.	*Theobroma grandiflorum* K. (Willd. ex Spreng.) Schum.	*Mangifera indica* L.	*Vitellaria paradoxa* C.F.Gaertn.	*Nephelium lappaceum* L.	*Bertholletia excelsa* Bonpl.	*Avena sativa* L.	*Jatropha curcas* L.	*Sevum Bos taurus* L.
64:37:1	63:34:3	54:42:3	53:41:7	47:46:5	51:48:1	26:39:31	20:40:39	22:40:36	43:43:5
Stearic	Stearic	Oleic	Oleic	Oleic	Oleic	Oleic	Oleic	Oleic	Oleic
48%	36%	41%	42%	46%	40%	39%	40%	40%	41%
Oleic	Oleic	Stearic	Stearic	Stearic	Arachidic	Linoleic	Linoleic	Linoleic	Palmitic
37%	34%	35%	40%	41%	35%	31%	38%	36%	23%
Arachidic	Palmitic	Arachidic	Palmitic	Linoleic	Stearic	Palmitic	Palmitic	Palmitic	Stearic
8%	25%	10%	9%	5%	7%	14%	18%	15%	15%
Palmitic	Linoleic	Palmitic	Linoleic	Palmitic	Palmitic	Stearic	Stearic	Stearic	Palmitoleic
7%	3%	9%	7%	5%	6%	11%	2%	7%	4%
	Arachidic	Linoleic	Arachidic	Arachidic	Gondoic		Linolenic	Linolenic	Myristic
	1%	3%	3%	1%	6%		1%	1%	3%
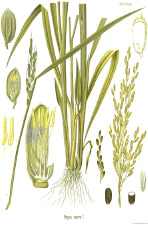	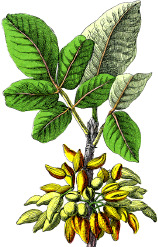	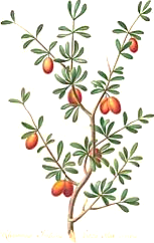	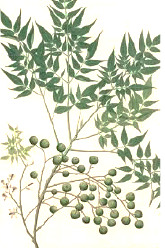	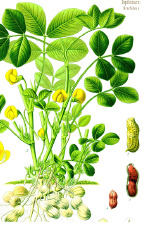	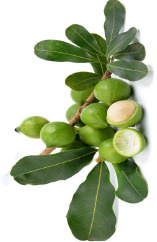	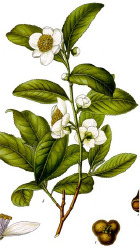	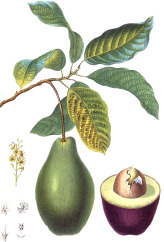	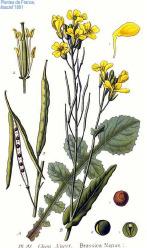	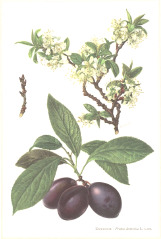
Rice bran	Pistachio nut	Argan fruit	Neem seed	Peanut bean	Macadamia nut	Tea seed	Avocado seed	Canola seed	Plum kernel
*Oryza sativa* L.	*Pistacia vera* L.	*Argania spinosa* (L.) Skeels	*Sideroxylon spinosum*. L Juss.	*Arachis hypogaea* L.	*Macadamia integrifolia* Maid & Bet	*Camellia sinensis* (L.) Kuntze	*Persea americana* Mill.	*Brassica napus* L.	*Prunus domestica* L.
21:42:36	26:47:27	18:46:36	34:46:18	18:50:31	15:80:4	19:58:22	16:68:15	8:62:32	7:65:27
Oleic	Oleic	Oleic	Oleic	Oleic	Oleic	Oleic	Oleic	Oleic	Oleic
42%	46%	46%	46%	48%	48%	58%	60%	60%	64%
Linoleic	Linoleic	Linoleic	Palmitic	Linoleic	Palmitoleic	Linoleic	Palmitic	Linoleic	Linoleic
35%	27%	34%	21%	30%	27%	22%	16%	22%	27%
Palmitic	Palmitic	Palmitic	Linoleic	Palmitic	Palmitic	Palmitic	Linoleic	Linolenic	Palmitic
18%	24%	12%	18%	10%	10%	15%	14%	10%	5%
Stearic	Stearic	Stearic	Stearic	Stearic	Linolenic	Stearic	Palmitoleic	Palmitic	Stearic
2%	2%	5%	13%	3%	3%	3%	7%	5%	1%
Linolenic	Palmitoleic	Linolenic		Arachidic	Vaccenic		Linolenic	Stearic	
1%	1%	1%		2%	3%		1%	2%	
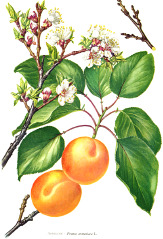	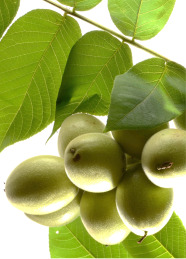	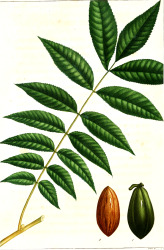	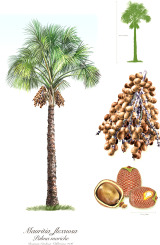	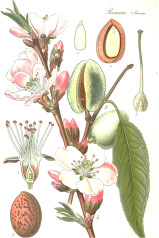	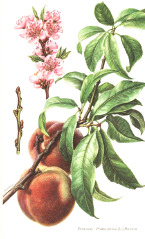	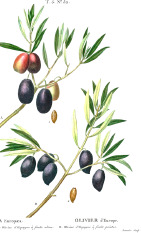	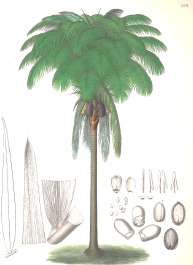	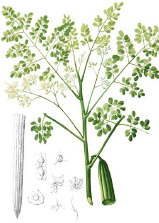	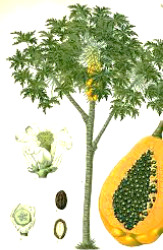
Apricot kernel	Marula fruit	Pecan nut	Buriti fruit	Almond nut	Peach kernel	Olive fruit	Pataua fruit	Ben seed	Papaya seed
*Prunus armeniaca* L.	*Sclerocarya birrea* (A.Rich.) Hochst.	*Carya illinoinensis* (Wangenh.) K. Koch	*Mauritia flexuosa* L.f.	*Prunus amygdalus* Batsch Webb	*Prunus persica* (L.) Batsch	*Olea europaea* L.	*Oenocarpus bataua* Mart.	*Moringa oleifera* Lam.	*Carica papaya* L.
5:66:29	35:68:6	8:68:24	19:69:12	8:71:20	9:74:18	15:74:10	20:76:3	19:80:1	19:79:3
Oleic	Oleic	Oleic	Oleic	Oleic	Oleic	Oleic	Oleic	Oleic	Oleic
66%	67%	68%	69%	70%	73%	73%	73%	78%	79%
Linoleic	Palmitic	Linoleic	Palmitic	Linoleic	Linoleic	Palmitic	Palmitic	Palmitic	Palmitic
29%	14%	22%	19%	20%	18%	12%	18%	7%	17%
Palmitic	Stearic	Palmitic	Linoleic	Palmitic	Palmitic	Linoleic	Vaccenic	Behenic	Linoleic
4%	9%	5%	11%	6%	6%	9%	2%	4%	3%
Stearic	Linoleic	Stearic		Stearic	Stearic	Stearic	Linoleic	Arachidic	Stearic
0.5%	6%	3%		1%	2%	3%	2%	4%	2%
		Linolenic						Stearic	
		2%						4%	
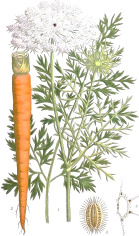	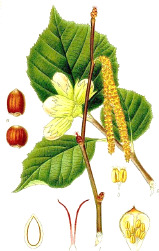	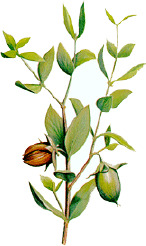	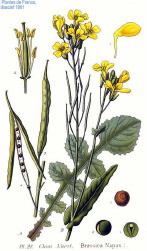	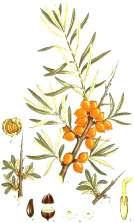	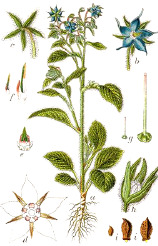	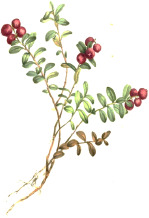	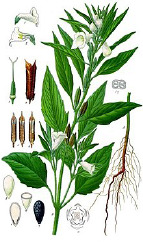	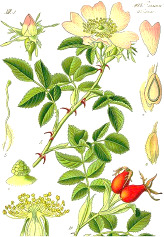	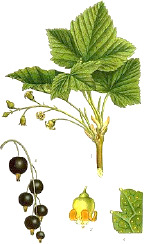
Carrot seed	Hazel nut	Jojoba seed	Mustard seed	Buckthorn seed	Borage seed	Cranberry seed	Sesame seed	Rosehip fruit	Currant seed
*Daucus carota* L.	*Corylus avellana* L.	*Simmondsia chinensis* (Link) C.K.Schneid.	*Sinapis alba* L.	*Hippophae rhamnoides* L.	*Borago officinalis* L.	*Vaccinium macrocarpon* Aiton	*Sesamum indicum* L.	*Rosa canina* L.	*Ribes nigrum* L.
5:82:13	8:83:9	1:98:1	9:75:16	11:22:67	17:26:61	10:23:68	16:40:46	6:12:82	8:16:75
Oleic	Oleic	Gondoic	Erucic	Linoleic	Linoleic	Linoleic	Linoleic	Linoleic	Linoleic
82%	83%	77%	51%	39%	39%	44%	45%	46%	48%
Linoleic	Linoleic	Erucic	Linoleic	Linolenic	g-Linolenic	Linolenic	Oleic	Linolenic	Oleic
13%	9%	12%	12%	29%	22%	22%	40%	31%	14%
Palmitic	Palmitic	Oleic	Oleic	Oleic	Oleic	Oleic	Palmitic	Oleic	g-Linolenic
4%	5%	9%	14%	19%	19%	23%	10%	12%	13%
	Stearic	Nervonic	Gondoic	Palmitic	Palmitic	Palmitic	Stearic	Palmitic	Linolenic
	3%	1%	9%	8%	12%	8%	5%	4%	12%
			Palmitic	Stearic	Stearic	Stearic			Palmitic
			4%	3%	4%	2%			6%
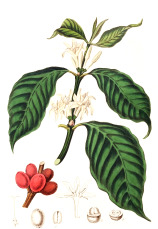	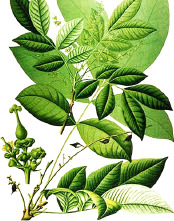	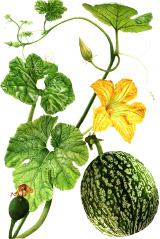	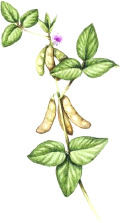	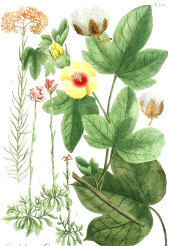	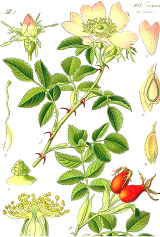	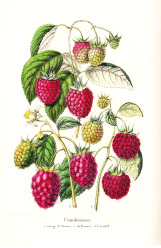	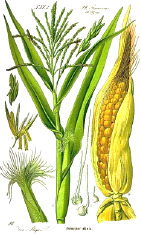	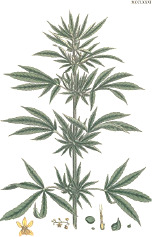	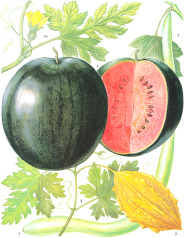
Coffee bean	Kusum seed	Pumpkin seed	Soybean seed	Cotton seed	Rosehip seed	Raspberry seed	Corn seed	Hemp seed	Watermelon seed
*Coffea arabica* L.	*Schleichera oleosa* (Lor.) Oken (Lour.) Merr.	*Cucurbita pepo* L.	*Glycine max* (L.) Merr.	*Gossypium arboretum* L.	*Rosa canina* L.	*Rubus idaeus* L.	*Zea mays* L.	*Cannabis sativa* L.	*Citrullus lanatus* (Thunb.) Matsum. & Nakai
44:7:49	9:36:7	22:26:52	16:24:60	26:18:53	6:20:75	4:12:84	15:28:57	10:12:79	22:18:60
Linoleic	Linolelaidic	Linoleic	Linoleic	Linoleic	Linoleic	Linoleic	Linoleic	Linoleic	Linoleic
48%	50%	51%	52%	52%	54%	55%	56%	56%	60%
Palmitic	Eicosenoic	Oleic	Oleic	Oleic	Linolenic	Linolenic	Oleic	Linolenic	Oleic
33%	30%	26%	24%	18%	19%	29%	28%	20%	18%
Stearic	Palmitic	Palmitic	Palmitic	Palmitic	Oleic	Oleic	Palmitic	Oleic	Palmitic
7%	8%	13%	11%	13%	20%	12%	12%	12%	11%
Oleic	Linoleic	Stearic	Linolenic	Stearic	Palmitic	Palmitic	Stearic	Palmitic	Stearic
7%	6%	9%	8%	12%	3%	3%	2%	6%	10%
Arachidic			Stearic						
3%			4%						
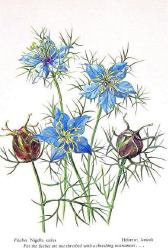	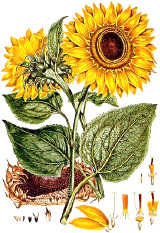	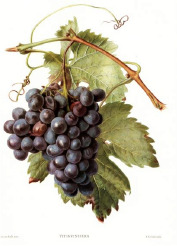	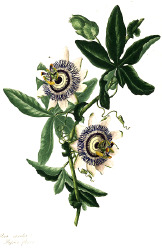	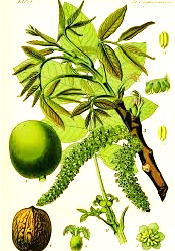	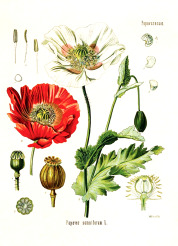	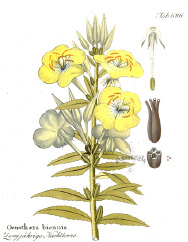	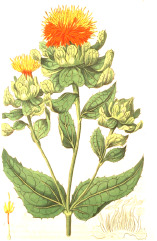	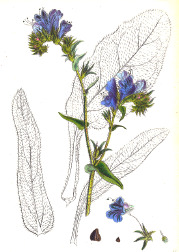	
Black cumin seed	Sunflower seed	Grape seed	Passion seed	Walnut seed	Poppy seed	Ev primrose seed	Safflower seed	Echium seed	
*Nigella sativa* L.	*Helianthus annuus* L.	*Vitis vinifera* L.	*Passiflora edulis* Sims	*Juglans regia* L.	*Papaver somniferum* L.	*Oenothera biennis* L.	*Carthamus tinctorius* L.	*Echium plantagineum* L.	
20:14:67	13:22:66	12:20:68	12:14:74	15:1:84	12:12:76	8:9:83	9:14:77	10:17:72	
Linoleic	Linoleic	Linoleic	Linoleic	Linoleic	Linoleic	Linoleic	Linoleic	Linolenic	
65%	66%	68%	73%	74%	74%	74%	76%	30%	
Oleic	Oleic	Oleic	Oleic	Linolenic	Oleic	g-Linolenic	Oleic	Linoleic	
14%	22%	20%	14%	10%	12%	10%	13%	19%	
Palmitic	Palmitic	Palmitic	Palmitic	Palmitic	Palmitic	Oleic	Palmitic	Oleic	
12%	6%	8%	10%	10%	10%	8%	6%	17%	
Lauric	Stearic	Stearic	Stearic	Stearic	Stearic	Palmitic	Stearic	Stearidonic	
4%	5%	4%	3%	4%	2%	6%	2%	13%	
						Stearic		g-Linolenic	
						2%		10%	
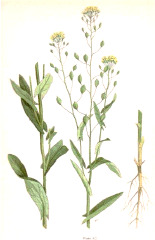	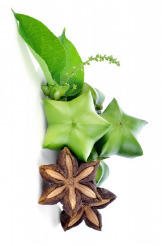	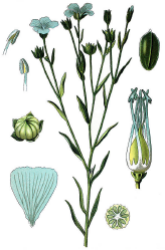	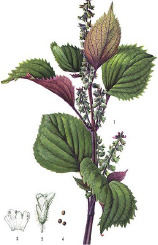	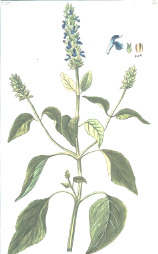	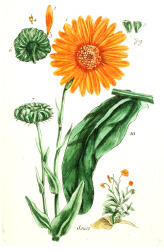	^1^ Botanical prints.			
*Camelina* seed	Sacha ichi seed	Flax seed	Perilla seed	Chia seed	Calendula seed	^2^ Common name, target plant tissue, and taxonomical name according to the Plants of the World database, Kewscience (2019).			
*Camelina sativa* (L.) Crantz	*Plukenetia volubilis* L.	*Linum usitatissimum* L.	*Perilla frutescens* (L.) Britton	*Salvia hispanica* L.	*Calendula officinalis* L.^2^				
10:33:54	7:8:84	10:19:72	12:18:70	10:7:81	7:6:87 ^3^	^3^ Saturation ratios are listed as saturated:momounsaturated:polyunsaturated fatty acids (%). The ratios may not add up to 100% due to rounding of percentages, as well as unidentified, unrecovered, or uncategorized constituents of oils—including, unsaponifiable fraction, trans-fats, phospholipids, and trace fatty acids. ^4^ Numbers were rounded to the nearest whole digit and means were used instead of ranges. Preference was given to recent original publications and those that utilized supercritical carbon dioxide extraction methods over organic solvents, as most suitable for cosmetic use.			
Linolenic	Linolenic	Linolenic	Linolenic	Linolenic	Calendic				
38%	50%	55%	55%	61%	57%				
Oleic	Linoleic	Oleic	Oleic	Linoleic	Linoleic				
19%	34%	18%	18%	20%	29%				
Linolenic	Oleic	Linoleic	Linoleic	Oleic	Oleic				
16%	8%	17%	15%	7%	5%				
Eicosenoic	Palmitic	Palmitic	Palmitic	Palmitic	Palmitic ^4^				
12%	4%	6%	9%	7%	4%				
Palmitic	Stearic	Stearic	Stearic	Stearic					
5%	3%	3%	3%	3%					

#### Palmitic Acid 16:0 and Stearic Acid 18:0

Palmitic acid 16:0 and stearic acid 18:0 are the most common saturated fatty acid esters in animal fat, often rendered as semisolid tallows used in a variety of traditional foods (shortenings, pemmican), salves, and ointments for irritated and inflamed skin conditions. All animal tallows are dominated by oleic acid 18:1(n-9) (from 70% in bear to from 26% in sheep fat), followed by palmitic acid 16:0 (from 28% in beef to 7% in bear fat) and stearic acid 18:0 (from 30% in goat to 3% in bear fat). Their saturation ratios vary from 69:29:2 in goat tallow, to 42:46:6 in pig lard, to 12:70:9 in bear tallow. Among botanical oils, palm pulp, sea buckthorn fruits, and cocoa beans contain naturally high amounts of palmitic acid 16:0. Cocoa beans oil also contains high levels of stearic acid 18:0, similar to kokum, sal, mango, and shea butters that is responsible for their semisolid appearance and consistency (**Table 4**). In human sebaceous glands, exogenously supplied palmitic acid 16:0 is desaturated at an unusual C6 position to produce sapienic acid 16:1(n-10), and both molecules can undergo elongation to stearic acid 18:0 and sebaleic acid 18:2(n-10), respectively. Both palmitic acid 16:0 and stearic acid 18:0 are preferred over palmitoleic acid 16:1(n-7) and oleic acid 18:1(n-9) for incorporation into wax esters, while linoleic acid 18:2(n-6) is the only fatty acid metabolized into two carbon precursors *via* β-oxidation in the skin (Pappas et al., 2002). The excess of palmitic acid 16:0 inhibits metabolism of linoleic acid 18:2(n-6) and α-linolenic acid 18:3(n-3) into the respective elongation products dihomo-γ-linolenic acid 20:3(n-6) and eicosatetraenoic acid 20:4(n-3), with the former one being a direct precursor of anti-inflammatory eicosanoids (Park et al., 2016).

#### Arachidic Acid 20:0, Behenic Acid 22:0, and Lignoceric Acid 24:0

Long chain saturated fatty acids with carbon chain length over C20 are present in skin in small quantities (**Tables 2** and **3**). In plants, moderate amounts of arachidic acid 20:0 can be found in rambutan, cupuacu, and peanut oils. Likewise, behenic acid 22:0 can be found in ben (moringa) and peanut oils, while lignoceric acid 24:0, a byproduct of plant lignin biosynthesis, can be found in wood tar and in minor quantities in peanut oil (**Table 4**). Despite their low bioavailability, these fatty acids are cholesterol-raising agents in humans (Cater and Denke, 2001), and therefore are used in topical skin and hair applications mostly for their lubricant and moisturizing properties.

### Unsaturated Fatty Acids

Unsaturated fatty acid esters present themselves as colorless liquids under normal conditions and are classified into monounsaturated (MUFA) and polyunsaturated (PUFA) fatty acids. They are further distinguished accordingly to the number and location of the double *cis* bonds in relationship to the carboxyl terminus as omega- (n-, delta-) 12, 11, 9, 7, 6, 5, and 3 fats. Two fatty acids, linoleic acid 18:2(n-6) and linolenic acid 18:3(n-3) are essential and must be obtained from the dietary sources. Additionally, many of plant secondary metabolites found in small amounts in unsaturated botanical oils have complimentary anti-inflammatory, antimicrobial, and antioxidant properties that can be also utilized manage clinical manifestations associated with skin damage or an immune disorder (Elshafie and Camele, 2017).

#### Oleic Acid 18:1(n-9) and Petroselinic Acid 18:1(n-12)

Oleic acid 18:1(n-9) is the most common botanical MUFA that is classified as omega-9. It is also the most common fatty acid of human adipose tissues (47–52%), followed by palmitic (22–25%), linoleic (11–13%), stearic (4–8%), palmitoleic (4–8%), and myristic (2–3%) acids (Kokatnur et al., 1979). Oleic acid triglycerides comprise the majority of botanical oils, including carrot seed, pataua, *Camellia* tea seed, papaya seed, marula, hazelnut, moringa, buriti, almond, plum, apricot, and peach kernels, olive, pistachio, canola, macadamia nut, avocado, peanut, mango seed, pecan, neem seed, argan, *Jatropha*, cupuacu, oat, brazil nut, and rice bran, to name a few (**Table 4**). High oleic (70–80%) cultivars of sunflower, safflower, and canola have been also developed as a primary source of dietary vegetable oils. Due to presence of a *cis* double bond, addition of oleic acid 18:1(n-9) to lipid bilayers increases instability and progressive structural loss (Akinshina et al., 2016). As such, oleic acid-based infusions, adjuvants, micelles, and vesicles are often used to penetrate the epithelium and enhance the topical delivery of drugs (Zakir et al., 2010).

Oleic acid 18:1(n-9) is toxic to keratinocytes when applied directly to the cells, and this effect is decreased in the presence of the functional epithelium barrier of the skin. Mild visible skin irritation and increased traffic of the inflammatory cells, combined with increased interleukin (IL)-1α and other cytokine production, have been also observed (Boelsma et al., 1996). Similarly, almond and avocado oils showed moderate skin and eye irritation when tested as pure oils and 2–10% aqueous emulsions in a subchronic 90-day repeated dose study in rabbit (Guillot et al., 1979). While dietary intake of olive oil has several beneficial effects on metabolic and skin health (Owen et al., 2000), its topical application is less effective due to its sensitizing or irritant effects (Kränke et al., 1997), and can be largely attributed to its polyphenol and squalene content. The beneficial effects of avocado oil on collagen metabolism (Werman et al., 1991), argan oil on cardiovascular health (Monfalouti et al., 2010), and oat lipids on keratinocyte differentiation (Chon et al., 2015) are also likely mediated by secondary bioactive compounds found in these oils.

Petroselinic acid is a naturally occurring positional isomer of oleic acid that belongs to omega-12 fats. Found in parsley *Petroselinum crispum* (Mill.) Fuss, coriander *Coriandrum sativum* L., and geranium *Geranium sanguineum* L. seed oils, it exhibits the disruptive tendencies in the lipid structures of the stratum corneum similar to those of oleic acid (Takeuchi et al., 1998).

#### Gondoic Acid 20:1(n-9), Erucic Acid 22:1(n-9), and Nervonic Acid 24:1(n-9)

These monounsaturated long chain fatty acids are omega-9 elongation products of oleic acid 18:1(n-9) found in small quantities in some botanical oils. *Camelina* seed oil is the major source of gondoic acid 20:1(n-9), while jojoba and mustard oils are the major sources of erucic acid 22:1(n-9) and nervonic acid 24:1(n-9) (**Table 4**). Similar to oleic acid, these molecules disrupt skin lipid layers and enhance permeation of drugs (Morimoto et al., 1996). Dietary supplementation with omega-9 fats can be also protective against metabolic risk factors associated with cardiovascular disease (Gillingham et al., 2011). In rare instances when β-oxidation of long chain saturated fatty acids is genetically disrupted, supplementation with long chain monenoic acid fatty acids can be beneficial to slow down demyelination (Sargent et al., 1994). However, dietary supplementation with erucic acid 22:1(n-9) induces alopecia and scaly skin lesions similar to those observed in essential fatty acids deficiency, suggesting that this fatty acid may interfere with dermal metabolism of essential fatty acids (Hulan et al., 1976).

#### Myristoleic 14:1(n-5), Palmitoleic 16:1(n-7), and Paullinic 20:1(n-7) Acids

These fatty acids are desaturation omega-5 and omega-7 products of the respective saturated fatty acids. Less common in nature, these fatty acids are found in small quantities in butters and animal fats. Among plant sources, nutmeg and saw palmetto oils contain measurable levels of myristoleic acid 14:1(n-5), while sea buckthorn and macadamia nut oils are a good source of palmitoleic acid 16:1(n-7) (**Table 4**). Paullinic acid 20:1(n-7) is rather uncommon, but it can be found in large quantities (40%) in guarana (*Paullinia elegans Cambess*.) seed oil, alongside another uncommon *cis*-vaccenic acid 18:1(n-7) (20%) (Spitzer, 1995). While generally regarded as anti-inflammatory, especially in the case of palmitoleic acid 16:1(n-7), their direct application to metabolic and immune health remains unclear (de Souza et al., 2018). Direct effects of palmitoleic-rich sea buckthorn oil (Hwang et al., 2012) or palmitoleic acid 16:1(n-7) alone (Weimann et al., 2018) on wound healing and skin aging was confirmed in the animal models, and sea buckthorn fruit oil (but not seed oil) was shown to improve symptoms of atopic dermatitis following oral supplementation with 5 g oil daily for 4 months to 49 subjects as a part of a placebo-controlled, double-blind clinical study (Yang et al., 1999).

#### Linoleic Acid 18:2(n-6)

Linoleic acid 18:2(n-6) is the most common botanical PUFA that is classified as an essential omega-6 fat. Large quantities of linoleic acid are found in evening primrose, safflower, sunflower, coffee beans, sesame, kusum (*trans*-linolelaidic acid), passion fruit seed, poppy seed, grape seed, watermelon seed, walnut, black cumin seed, hemp, raspberry seed, cotton seed, corn, soybean, pumpkin seed, rosehip, black currant seed, borage, cranberry seed, and sea buckthorn seed oils (**Table 4**). In humans, it serves as a starting point for biosynthesis of long-chain PUFAs, such as γ-linolenic acid 18:3(n-6), dihomo-γ-linolenic acid 20:3(n-6), and arachidonic acid 20:4(n-6), the latter being the major precursor to prostaglandins, leukotrienes, and endocannabinoids. Linoleic acid 18:2(n-6) is also selectively targeted for β-oxidation in the sebaceous gland to synthesize squalene and wax esters. Low levels of linoleic acid 18:2(n-6) also impair the epidermal barrier function and increase permeability of comedonal wall (Cunliffe et al., 2004). Consequently, sunflower oil high in linoleic acid 18:2(n-6) better preserves lipid integrity, does not cause erythema, and improves skin hydration in contrast to application of olive oil (Danby et al., 2013). However, many randomized controlled trials performed with evening primrose (Bamford et al., 2013) or borage (Foster et al., 2010) oils showed only minor to no beneficial effects on skin health outcomes, suggesting that minor components of linoleic-rich oils or increased proportion of oleic acid in these and other high linoleic oils could be partially responsible for these observations.

In this regard, hemp oil stands out among the linoleic-rich oils by having relatively low oleic 18:1(n-9) and high α-linolenic 18:3(n-3) content. Dietary hempseed oil improved skin dryness, itchiness, and decreased dermal medication use in a 20-week randomized crossover clinical study with atopic dermatitis patients (Callaway et al., 2005). Furthermore, a nonpsychotropic phytocannabinoid, cannabidiol, naturally present in hemp oil, showed some evidence of sebostatic actions like decreasing lipolysis, keratinocyte differentiation, and immune skin cell activation (Oláh et al., 2014).

#### γ-Linolenic 18:3(n-6), Dihomo-γ-Linolenic 20:3(n-6), Arachidonic 20:4(n-6), and Calendic 18:3(n-6) Acids

**Figure 1:**
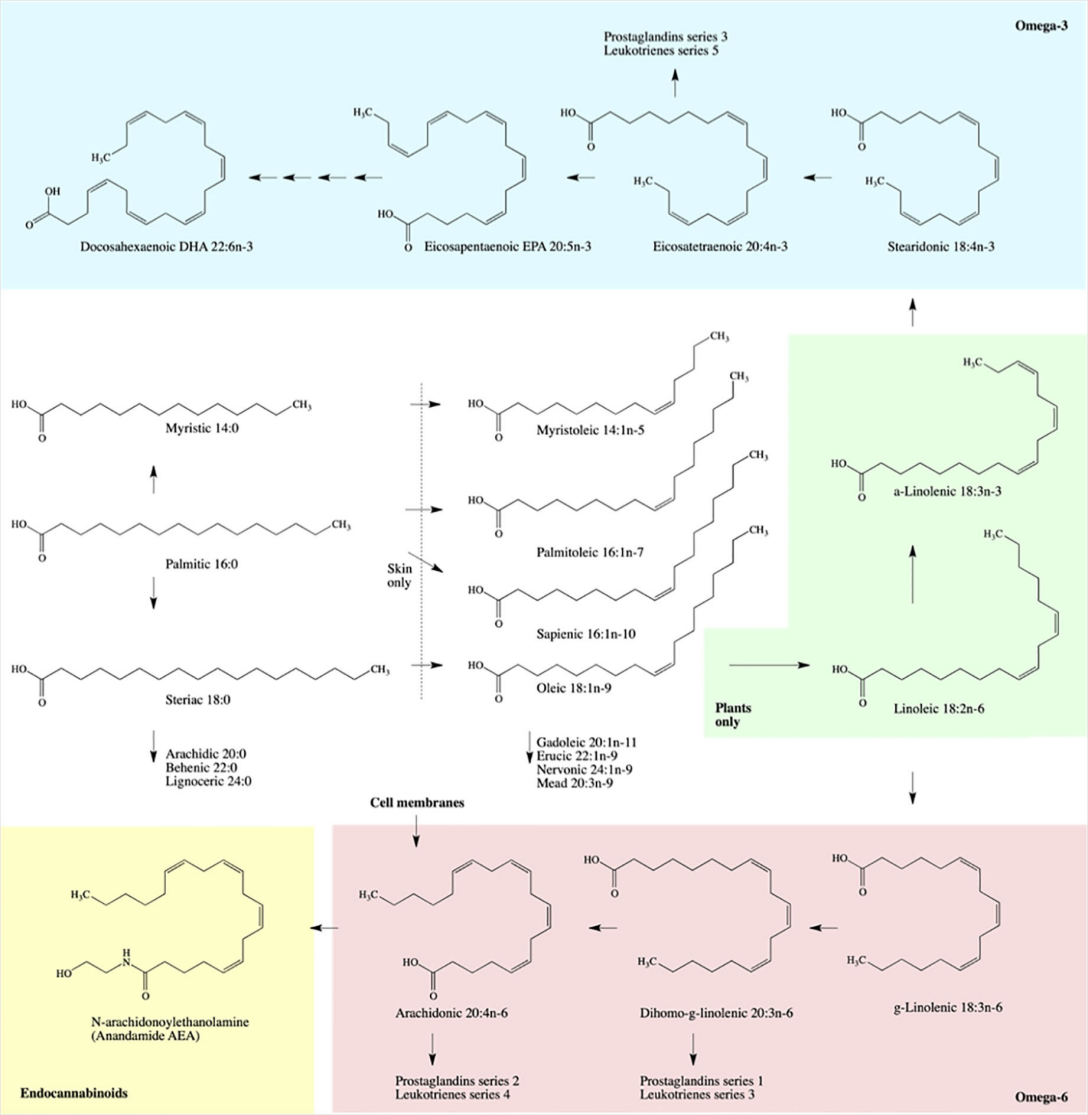
Schematic biosynthesis pathways of fatty acid metabolism.

γ-Linolenic acid 18:3(n-6) is found in several botanical oils including borage, black currant, and evening primrose (**Table 4**). Although generally considered anti-inflammatory, its clinical effectiveness for rheumatoid arthritis and skin conditions is questionable (Sergeant et al., 2016). Upon absorption, γ-linolenic acid is rapidly converted to dihomo-γ-linolenic acid 20:3(n-6) and stored in cellular glycerolipids of the immune (neutrophils) and skin cells, while it is converted both to dihomo-γ-linolenic acid and pro-inflammatory arachidonic acid 20:4(n-6) in liver and systemic circulation. Combination of γ-linolenic acid supplementation with omega-3 fatty acid such as docosahexaenoic acid (DHA) or Environmental Protection Agency (EPA) (discussed in *Docosahexaenoic 22:6(n-3), Eicosapentaenoic 20:5(n-3), and Stearidonic 18:4(n-3) Acids*), however, seem to preferentially inhibit conversion of γ-linolenic acid to arachidonic acid (Barham et al., 2000) and improve immune outcomes in part by modulating the ratios of series 2 *versus* series 1 prostaglandins (**Figure 1**). This effect can be achieved directly by a combined supplementation of γ-linolenic botanical oils (such as borage) and omega-3 enriched botanical oils such as hemp or echium (Lee et al., 2014). Arachidonic acid is also a major source of oxidized bioactive lipid mediators that stimulate proliferation, migration, and homing of cells in the wound bed and promote early stages of wound healing (Dhall et al., 2015).

Calendic acid 18:3(n-6) is a positional isomer of linoleic acid found in significant quantities in seeds of pot marigold (Dulf et al., 2013). Similar to other conjugated linolenic acids, supplementation with calendic acid improved metabolic risk factors (Chardigny et al., 2003), however its relationship to skin health has not been established.

#### α-Linolenic 18:3(n-3) and Punicic 18:3(n-5) Acids

α-Linolenic acid is an essential omega-3 fatty acid found in multiple seed oils, such as chia, perilla, flax, sacha inchi, *Camelina*, sea buckthorn, cranberry, rosehip, black currant, raspberry, and hemp (**Table 4**). This acid is a minor physiological component of cellular and mitochondrial membranes that regulates cell signaling and transport across the lipid bilayers. Both α-linolenic acid and linoleic acids reduce UV-associated damage and hyperpigmentation of the skin (Ando et al., 1998; Barham et al., 2000). Dietary α-linolenic acid can be slowly metabolized into eicosapentaenoic acid 20:5n-3 and docosahexaenoic acid 22:6n-3, however with the efficiency of only a few percent (Burdge and Calder, 2005). The bulk of α-linolenic acid metabolites, however, is used to synthesize anti-inflammatory series of prostaglandins and leukotrienes.

A topical formulation containing 4% chia seed oil applied for 8 weeks improved skin hydration and subjective itching in subjects with pruritus and xerosis (Jeong et al., 2010). A randomized, double-blind 12-week supplementation with flax seed oil increased smoothness and hydration of the skin, while reducing skin irritation and scaling following the nicotinate challenge (Neukam et al., 2011). Two months supplementation with black currant seed oil also enhanced the skin immune response and reduced prostaglandin E(2) production (Wu et al., 1999). Sacha inchi and rosehip oils high in α-linolenic acid showed no toxicity toward keratinocytes and moderate inhibition of *Staphylococcus aureus* adherence in cell culture (Gonzalez-Aspajo et al., 2015) and promoted wound healing by improving scarring outcome and macrophage phenotypes transition (Lei et al., 2018). Punicic acid, a conjugated positional isomer of α-linolenic acid at *cis*-9, *trans*-11, and *cis*-13 positions found in high amounts in pomegranate seed oil, also enhanced transdermal delivery of resveratrol (Liu et al., 2018).

#### Docosahexaenoic 22:6(n-3), Eicosapentaenoic 20:5(n-3), and Stearidonic 18:4(n-3) Acids

Long chain PUFA such as docosahexaenoic acid DHA 22:6(n-3), and eicosapentaenoic acid EPA 20:5(n-3) found in abundance in marine fish and krill oils are produced by various types of marine algae and accumulate in these animals as a part of their alga-based diet. As various alga have a different capacity to synthesize DHA or EPA, the compositions of marine oils vary considerably upon fish species, diet, body part, extraction method employed, and typically lack significant amounts of long chain PUFA when derived from freshwater sources (Mohanty et al., 2016). Their various metabolites are essential in the regulation of inflammation and immune outcomes, similar to their precursors α-linolenic and stearidonic acids (McCusker and Grant-Kels, 2010). While DHA is predominantly found in the brain and retina, EPA shows direct competition with arachidonic acid metabolic pathways, thus reducing pro-inflammatory eicosanoids levels and suppressing inflammation (Lorente-Cebrián et al., 2015).

In the skin, both DHA and EPA reduce UVB damage and IL-8 secretion (Storey et al., 2005), and this effect can be achieved by topical application of 10 μmol DHA by upregulation of Nrf2 and heme oxygenase-1 signaling (Yum et al., 2017). A larger dose of DHA (30 μM) was also effective to promote wound healing (Arantes et al., 2016), although another study suggested a following order of potency in modulating skin wound healing: omega-9 > omega-6 > omega-3 (Cardoso et al., 2004).

Stearidonic acid 18:4(n-3) is a metabolic precursor to DHA and EPA that can be found in certain botanical oils like hemp, black currant, echium, and spirulina (**Table 4**). While only 5–20% and 1–9% of α-linolenic acid is converted to EPA and DHA in humans, it is estimated that stearidonic acid is much more efficient substrate (17–40%) for such a conversion (Whelan, 2009). Its direct relationship to skin health has not been established.

## Potential Toxicities Associated with Botanical Oils

Botanical oils contain complex mixtures of both saturated and unsaturated fatty acids, typically esterified in the form of triglycerides, that act as powerful lipophilic solvents to selectively extract and accumulate nonpolar secondary metabolites produced by the source plants. Although data is limited, the most efficient solvent-like molecules in the fixed (oleic and linoleic acids) and essential (limonene) botanical oils are likely of low toxicity. However, these compounds may cause mild skin irritation, while their oxidation products may produce dermal sensitization in humans (DeWitt and Bebarta, 2004). The potentially detrimental effects may also vary with dose, form (water or fat soluble), and site of application (sebaceous gland, hair follicles), which will define absorption rates and accumulation of the bioactive compounds in various layers of the skin and systemic circulation.

Most of botanical oils are generally well tolerated in adults, with occasional allergic skin reactions occurring, and are “generally recognized as safe” (GRAS) as a food (dietary supplement) by the U.S. Food and Drug Administration. This designation, however, does not require the manufacturer to prove the safety and efficacy of the product prior to marketing. Botanical oils produced by different manufacturers may also contain different ingredients that do not match the actual ingredients or their amounts listed on the label. As such, botanical oils are considered as alternative (complimentary) strategies used to supplement the perceived failures and side effects of conventional medicines.

### Site of Application

Regional permeability of the human body is not uniform and is typically ranked as follows: scrotum > face/scalp > trunk/extremities > palm/sole > nail. Within those regions, further variations in stratum corneum thickness, the number of sebaceous glands, and hydration status can all affect absorption and metabolism of botanical oils and their bioactive components. Understanding the parameters that affect the permeability of the skin barrier is essential for achieving correct dosing and adherence regimens with a goal of reaching therapeutic targets within the local skin environment (ointment or cream) or systemic uptake *via* dermal capillary beds (Prausnitz and Langer, 2008). The use of botanical oils as vehicles for therapeutic drug delivery provides a wide variety of choices between achieving optimal drug potency and therapeutic effectiveness, as well as the risk of over-added toxicity, as the same drug may appear in different potency classes when formulated in different vehicles or applied to different target site (Williams and Barry, 2004).

### Genotoxicity and Photosensitivity

Some of secondary metabolites coextracted with botanical oils may form genotoxic DNA adducts or activate detoxification enzymes, as it was shown for safrole and quinones in sassafras oil or epoxides found in pennyroyal oil (Dietz and Bolton, 2011). Other botanical oils and their constituents may exhibit a dual genotoxic and antigenotoxic effect, as it was shown for β-caryophyllene (Di Sotto et al., 2010). Adverse cutaneous responses to the combined action of the botanical oil or its bioactive constituent and UV radiation may cause phototoxic reactions that result in sunburn, edema, hyperpigmentation, photoaging, and cancer (Gould et al., 1995). Some of these effects, however, may be beneficial in alleviating multiple symptoms of psoriasis, vitiligo, and cutaneous T-cell lymphoma (Bark et al., 2010).

### Secondary Metabolites and Biological Reactive Intermediates

While most botanical oils can be considered safe, a few contain compounds, which can be converted to biological reactive intermediates causing toxicity (Llana-Ruiz-Cabello et al., 2015). Although health promoting effects of secondary metabolites coextracted into the botanical oils may be beneficial, they may also have potential toxic effects and local higher levels of exposure due to topical application. For example, rosemary oil has been demonstrated to induce lipid and protein oxidation at high doses (Estévez and Cava, 2006). High doses of the monoterpenoid phenols, carvacrol, and thymol, increase the levels of malondialdehyde, resulting in membrane damage, and 8-hydroxy-deoxyguanosine, causing cell DNA damage (Ozkan and Erdogan, 2012). Eugenol present in clove oil can be oxidized to phenoxyl radicals that induce reactive oxygen species-mediated apoptosis in human cells (Yoo et al., 2005). Borage plant parts contain pyrrolizidine alkaloids that are toxic to the liver and lungs, and maybe coextracted into borage seed oil (Low Dog, 2009). Raw botanical oil materials often originate from different sources and storage timeframes, complicating comparisons of bioactive ingredients and lack of potentially toxic contaminants in them.

### Overdose in Pregnant Women and Children

In rare instances, some commercially marketed hemp seed oils could lead to mild cannabinoid poisoning in children (Chinello et al., 2017) and pregnant women (Yang et al., 2017). While food-grade strains of hemp must contain less than 0.3% tetrahydrocannabinol (THC) by weight (whole plant), hemp seeds, or stems used to produce hemp oil may become contaminated by THC-rich trichomes of hemp flowers and thus acquire THC (Yang et al., 2017). Due to the polymorphic nature of cytochrome P450 enzymes that can be further affected by age, liver impairment, or potential drug interactions, people consuming hemp products may gradually accumulate THC due to its slow metabolism or relatively long half-life in the body, resulting in potentially higher concentrations (Watanabe et al., 2007).

### Neonatal Skin Sensitization

Infant skin is susceptible to dryness and irritation from external factors, including topical skin care products not formulated for the infant’s skin (Kuller, 2016). Topical products with adverse effects on skin barrier function, however, carry a potential to develop atopic dermatitis or eczema (Danby et al., 2013). The practice of recommending and using topical oils for the prevention or treatment of baby dry skin or for massage, including the increased societal interest in natural interventions, often ignores the fact that specific topical oils may have an adverse effect on skin barrier function (Cooke et al., 2011). While oils with the lowest oleic acid content provide a lower risk of irritant contact dermatitis (Kuller, 2016), sunflower-based oils may also may retard postnatal skin barrier maturation in infants (Kanti et al., 2014). Skin ointments containing components of food origin also carry the risk of possible percutaneous sensitization to food proteins that may promote development of contact dermatitis and persistent eczema, as it was shown for almond oil (Guillet and Guillet, 2000).

## Conclusions

Both topical and dietary interventions with botanical oils may produce different functional outcomes according to their phytochemical composition and the pathophysiological state of the target tissue. The depletion or disturbance of any of the skin lipid classes results in a rapid disruption of skin integrity, and leads to a variety of structural (barrier), physiological (repair and regeneration), and pathological (inflammation) changes that allow further entry of microbial and chemical irritants and deterioration of the affected, aged, or diseased skin. Replenishment of those lipids by direct replacement or enhancement of their *in situ* production with botanical oils may restore skin function and reduce pathophysiological symptoms associated with the disease. The botanical oil sources were arranged in clusters that reflect their relative fatty acid compositions and allow for easy substitution or replacement strategies to reformulate or develop novel functional interventions in skin care.

Among inexpensive, widely available oils, sunflower oil high in the omega-6 linoleic acid, and flax or hemp oil enriched with the omega-3 α-linolenic acid, offer an attractive combination of enhanced metabolic and reduced inflammatory and comedogenic effects. On the other hand, application of olive oil with high oleic acid content is warranted when deep transdermal penetration is desired, and the target skin site can be further sealed off with the application of highly saturated coconut or shea butters. The presence of dissolved bioactive secondary metabolites that target a specific health outcome will further substantiate the use of a particular plant source within the group of botanical oils with similar physiochemical properties.

To become established in clinical settings, the required mixtures and doses should be individually determined in randomized controlled trials that simultaneously monitor for hazardous effects of botanical oil supplementation to reduce the possible over-added toxicity associated with the interventions.

## Author Contributions

EM drafted and contributed knowledge on skin structure, disorders, toxicology and lipid compositions associated with healthy and disease skin states. EM and CW drafted and contributed knowledge on botanical oils composition and use. EM and SK conceived, designed, and wrote the manuscript, CW edited the manuscript. All authors read and approved the manuscript.

## Funding

This work was supported in part by NCSU faculty start-up funds (SK).

## Conflict of Interest

The authors declare that the research was conducted in the absence of any commercial or financial relationships that could be construed as a potential conflict of interest.
